# Radial Glia and Neuronal-like Ependymal Cells Are Present within the Spinal Cord of the Trunk (Body) in the Leopard Gecko (*Eublepharis macularius*)

**DOI:** 10.3390/jdb10020021

**Published:** 2022-06-01

**Authors:** Sarah V. Donato, Matthew K. Vickaryous

**Affiliations:** Department of Biomedical Sciences, Ontario Veterinary College, University of Guelph, Guelph, ON N1G 2W1, Canada; sdonato@uoguelph.ca

**Keywords:** lizard, neurobiology, central nervous system, neural stem cell, spinal cord

## Abstract

As is the case for many lizards, leopard geckos (*Eublepharis macularius*) can self-detach a portion of their tail to escape predation, and then regenerate a replacement complete with a spinal cord. Previous research has shown that endogenous populations of neural stem/progenitor cells (NSPCs) reside within the spinal cord of the original tail. In response to tail loss, these NSPCs are activated and contribute to regeneration. Here, we investigate whether similar populations of NSPCs are found within the spinal cord of the trunk (body). Using a long-duration 5-bromo-2′-deoxyuridine pulse-chase experiment, we determined that a population of cells within the ependymal layer are label-retaining following a 20-week chase. Tail loss does not significantly alter rates of ependymal cell proliferation within the trunk spinal cord. Ependymal cells of the trunk spinal cord express SOX2 and represent at least two distinct cell populations: radial glial-like (glial fibrillary acidic protein- and Vimentin-expressing) cells; and neuronal-like (HuCD-expressing) cells. Taken together, these data demonstrate that NSPCs of the trunk spinal cord closely resemble those of the tail and support the use of the tail spinal cord as a less invasive proxy for body spinal cord injury investigations.

## 1. Introduction

In mammals, the intrinsic ability to repair the spinal cord is limited [1,2,3]. Although there are currently no curative treatments, numerous efforts have focused on the potential of neural stem/progenitor cells (NSPCs) [4,5,6] and how these cells may be endogenously recruited to promote the re-acquisition of function [7]. Crucial insight into the potential of resident NSPCs to stimulate and facilitate repair comes from comparative studies involving various non-mammalian species including zebrafish, axolotls, and, increasingly, lizards [8,9,10,11,12,13,14]; see also [15].

For many lizards, tail regeneration is associated with a naturally evolved mechanism that closely parallels surgical amputation: tail autotomy. Tail autotomy is the ability to self-detach part of the tail to avoid or escape predation (e.g., [16]). The result is a complete rupture of multiple tissue types, including the spinal cord [10]. Previous investigations have revealed that, following tail autotomy, ependymal cells of the remaining spinal cord proliferate and create the regenerated spinal cord (e.g., [10,11,17,18]; reviewed in [19]).

Spontaneous regeneration of the spinal cord following injury involves the activation of an endogenous pool of NSPCs. The primary source of NSPCs is the ependymal layer lining the central canal [4,5]. In lizards and other non-mammalian species, NSPCs of the ependymal layer are typically identified as radial glia [10,20,21,22] (=ependymal radial glia, ependymoglia, or ependymo-radial glia; [23,24,25]). Radial glia are typically defined as a population of stem-like cells with astrocytic/astroglial characteristics, and an elongate basal (radial) process that spans from the ventricular lumen to the pial surface [21,26,27,28]. Characteristically, radial glia express a conserved panel of protein markers. These include the pluripotency transcription factor SOX2, and the intermediate filaments glial fibrillary acidic protein (GFAP) and Vimentin. In addition, NSPCs are known to be slow-cycling or quiescent under physiologically normal conditions (i.e., prior to injury; [12,29,30]). NSPC quiescence has been suggested as a mechanism to preserve DNA integrity [31]. However, following injury, rates of proliferation markedly increase [10,12,32]; see also [18].

While radial glia have been identified within the tail spinal cord, less is known about the trunk (body) spinal cord. Here, we characterize ependymal cells from the trunk spinal cord of the leopard gecko (*Eublepharis macularius;* hereafter ‘gecko’), a lizard capable of tail (and tail spinal cord) regeneration [20]. We hypothesized that ependymal cells from the trunk spinal cord includes populations of radial glia, and that these cells are activated in response to tail autotomy. We determined that ependymal cells of the trunk spinal cord represent a heterogenous assemblage, including both radial glia and a neuronal-like population. While trunk ependymal cells proliferate under homeostatic conditions, we found no evidence that rates significantly increased following tail loss. Although the in situ regenerative capacity of these cells remains poorly understood (although see [15]), our data reveal that ependymal cells of the trunk spinal cord are essentially identical to those in the tail, suggesting that the regenerative capacity of the central nervous system may extend into the body proper.

## 2. Materials and Methods

### 2.1. Animal Care

Juvenile, captive-bred leopard geckos (*Eublepharis macularius*) were obtained from a commercial supplier (Global Exotic Pets, Kitchener, ON, Canada). The University of Guelph approved Animal Utilization Protocol 1954 which complies with Canadian Council on Animal Care procedures and policies. Animal husbandry requirements were followed as described in McLean and Vickaryous [20]. Briefly, leopard geckos (ranging in mass from 8.0–34.4 g) were housed in the Hagen Aqualab, University of Guelph, in 5-gallon polycarbonate tanks inside a controlled environmental chamber with a 12:12 photoperiod and ambient temperature of ~27 °C. Using a heating cable (Hagen Inc., Baie d’Urfé, QC, Canada) set at 32 °C, heat was provided to one end of each enclosure (situated atop the cable) to establish a thermal gradient. Geckos had constant access to water, replaced a minimum of twice weekly, and were fed 2–3 mealworms per day. Mealworms were gut-loaded and dusted with powdered calcium and vitamin D3 (cholecalciferol) for dietary supplementation (Zoo Med Laboratories Inc., San Lupis Obispo, CA, USA). Health status was assessed weekly with mass and length measurements (snout-to-vent length, tail length, regenerate tail length). A total of 21 geckos were used for this study. We used 6 geckos for each of the long and short-duration 5-bromo-2′-deoxyuridine (BrdU) experiments (=12 BrdU geckos). For the protein expression characterization we used 12 geckos, three of which were also used for the short-duration BrdU experiment.

### 2.2. Tail Autotomy

Lizards capable of tail autotomy are able to voluntarily self-detach their tails in an effort to escape predation. Tail autotomy was used to induce tail loss by manually restraining the geckos and firmly pinching the tail between forefinger and thumb until the tail is shed. Following tail loss, geckos were returned to their enclosures and allowed to undergo wound healing and subsequent regeneration before being collected for tissue preparation and analysis.

### 2.3. 5-Bromo-2′-Deoxyuridine (BrdU) Pulse-Chase Experiments

To label proliferating cells, we used 5-bromo-2′-deoxyuridine (BrdU). BrdU is a thymidine analogue that is incorporated into DNA as a cell undergoes the synthesis phase of the cell cycle. The timeframe during which BrdU is administered is referred to as the pulse period. Any cells that cycle during the pulse period take up the analogue, which can then be detected using immunofluorescence. BrdU was injected into the peritoneal cavity of each experimental gecko at a dose of 50 mg/kg [stock solution diluted to 50 mg/mL in dimethyl sulfoxide, and working solution diluted to 5 mg/mL in sterile, injectable phosphate buffered saline (PBS)] twice daily, using a 0.5 cc insulin syringe (Abbott Laboratories, Saint-Laurent, QC, Canada). Injections alternated between the left side of the peritoneal cavity and the right side of the peritoneal cavity.

To identify quiescent cells, we conducted a long-duration (20 week) BrdU pulse-chase experiment. Geckos with original tails were pulsed with BrdU for seven (7) days (twice daily), and were then divided into two collection groups: immediately following the pulse (0 days chase; *n* = 3), and 20-weeks post-pulse (140-day chase; *n* = 3). BrdU was not administered during the 140-day chase period.

To determine whether ependymal cells constitutively proliferate and investigate if cell proliferation is affected by tail loss, we conducted a short-duration BrdU experiment using two groups of geckos. Geckos were assigned to one of two conditions: constitutive proliferation (original-tailed geckos), or proliferative response to tail loss. Geckos in the tail loss condition were stimulated to autotomize (= day 0). Immediately following autotomy, both original tail and tail loss groups were pulsed with BrdU for two days (twice daily). Geckos from both conditions were sampled immediately following the pulse (0 days chase; *n* = 6).

### 2.4. Tissue Collection and Preparation

Geckos were euthanized with an intra-muscular injection of Alfaxan (Alfaxalone; 85 mg/kg), then exsanguinated and fixed via transcardial perfusion of PBS followed by 10% neutral buffered formalin (NBF; Fisher Scientific, Waltham, MA, USA). Tissues were then immersed in NBF for an additional ~22 h to complete fixation and subsequently stored in 70% ethanol.

**Figure 1:**
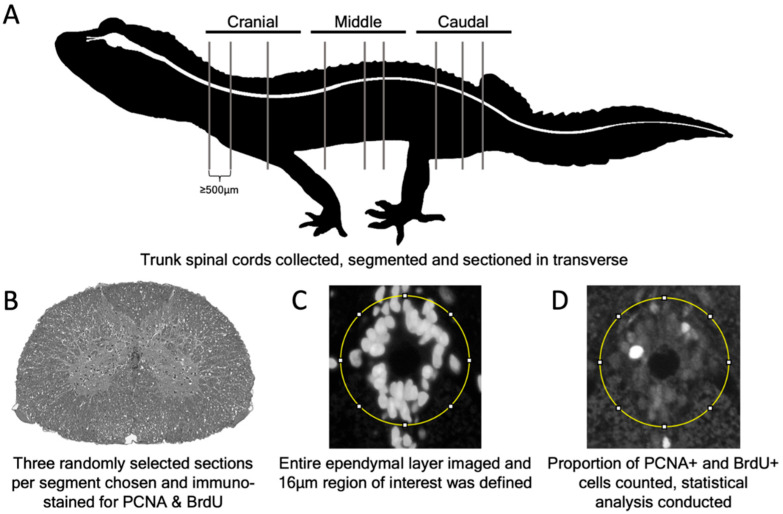
Experimental design. (**A**) Schematic representation of the leopard gecko (*Eublepharis macularius*) in lateral view. The central nervous system is highlighted in white. Trunk spinal cords (*n* = 3 per group) were collected, divided into three equal segments (cranial, middle and caudal), and sectioned in the transverse plane. (**B**) Three random sections, 500 to 2500 µm apart, were selected and immunostained for either PCNA or BrdU. (**C**) The entire ependymal layer was then imaged. To define the region of interest, a 16 µm diameter circle was drawn around the ependymal layer, originating from the center of the central canal. (**D**) The proportion of PCNA+ or BrdU+ cells across each group was collected using ImageJ and statistically analyzed.

To characterize trunk spinal cord ependymal cells, we sampled tail regenerating geckos at three different time points (2 days post-tail loss; 8 days post-tail loss; and 12 days post-tail loss), as well as original tailed geckos. We defined the trunk as the region of the body located between the cranial margin of the pectoral apparatus and the caudal margin of the pelvic apparatus (Figure 1A). To isolate the trunk region of the spinal cord, we removed the limbs, tail, and head/neck, followed by the pectoral apparatus, the ventral portion of the pelvic apparatus, and the organs of the coelomic cavity. The remaining vertebral column (including spinal cord) and associated musculature was then subdivided into three equal segments, termed cranial, middle and caudal (Figure 1A).

To prepare for serial histology, the segments of tissue were decalcified for 30 min in Cal-Ex^®^ (Fisher Scientific, Waltham, MA, USA) prior to processing. Tissue segments were dehydrated in 100% isopropanol, cleared in xylene and infiltrated with paraffin wax using an automated processor (Fisher Scientific, Waltham, MA, USA) prior to being embedded in paraffin wax blocks. Using a rotary microtome (HM 355S Automatic Microtome; Thermo Scientific, Ottawa, ON, Canada), tissues were then sectioned at 5 µm and mounted on charged slides (Surgipath X-Tra; Leica Microsystems, Ontario, BC, Canada) prior to baking at 60 °C overnight.

### 2.5. Hematoxylin and Eosin

For histological examination of the trunk spinal cord, representative serial sections were stained with hematoxylin and eosin. Slides were deparaffinized in three washes of xylene (2 min each), and rehydrated to water through three washes of 100% isopropanol (2 min each) and one wash of 70% isopropanol (2 min) before being brought to deionized water (dH_2_O; 2 min). Slides were then immersed in modified Harris hematoxylin (Fisher Scientific, Waltham, MA, USA) for 10 min, rinsed in running dH_2_O, and dipped six times in acid alcohol (1% hydrochloric acid in 70% isopropanol) to differentiate. Slides were then rinsed in dH_2_O, dipped in ammonia water until blue (~4 dips), and rinsed again in dH_2_O. Next, slides were dipped six times in 70% isopropanol prior to staining with eosin for 1 min. Slides were then brought through three washes of 100% isopropanol (2 min each) and cleared in three washes of xylene (2 min each) before being coverslipped with Cytoseal (Fisher Scientific, Waltham, MA, USA).

### 2.6. Immunofluorescence

The primary antibodies used and their associated protocols are listed in Table 1. Primary antibodies had previously been validated for use in the leopard gecko [10]. Omission controls lacking primary antibody were used to confirm the specificity of the secondary antibody. All omission controls were negative for immunostaining.

To identify radial glia, our investigation focused on three classic markers: SOX2, glial fibrillary acidic protein (GFAP) and Vimentin. SOX2 is one of the original four Yamanaka pluripotency factors [33] and participates in the maintenance of neural progenitor populations [34]. Both GFAP and Vimentin are intermediate filaments. In mammals, Vimentin is characteristic of neuroepithelial cells; the embryonic precursors of ependymal cells. However, following development of the spinal cord, the Vimentin content of these cells is gradually replaced by GFAP [35]. In contrast, among species capable of spinal cord regeneration, such as zebrafish, Vimentin expression is typically maintained by ependymal cells throughout adulthood [34,36].

To establish if cells of the ependymal layer constitutively proliferate, we investigated all three segments of the trunk spinal cord prior to tail loss. To estimate cell proliferation, we used two protein markers—proliferating cell nuclear antigen (PCNA), an S phase marker, and phosphor Histone H3 (pHH3), an M phase marker [37,38]—and conducted a short-duration (2 day) BrdU experiment (see Section 2.3).

To identify neuronal-like populations within the ependymal layer of the trunk spinal cord, we immuostained for two neuronal proteins: HuCD and Neuronal Nuclei (NeuN). HuCD is a pan-neuronal marker and is reportedly expressed by neuronal-like cells of the ependymal layer known as cerebrospinal fluid-contacting (CSF-c) cells [39,40,41,42]. Similar to neurons, CSF-c cells have large, round nuclei [43] and some are even capable of firing action potentials [44]. NeuN is a marker specific to the majority of mammalian neurons, including those of the gecko tail spinal cord [10].

#### 2.6.1. Standard Immunofluorescence Protocol

Standard immunofluorescence was used to detect PCNA and pHH3. Sections were rehydrated to dH_2_O (see above) prior to either antigen retrieval (pHH3 only; slides immersed in citrate buffer heated to 95 °C for 12 min, followed by a 20-min cool at room temperature) followed by three washes of PBS for 2 min each, or three washes of PBS (2 min each) if citrate buffer retrieval was not required (PCNA only; see Table 1). Sections were then blocked with 3% normal goat serum (NGS; Vector Laboratories, Burlingame, CA, USA) diluted in sterile PBS for one hour at room temperature before being tipped off and incubated overnight with primary antibody (rabbit anti-PCNA [1:100]; Santa-Cruz Biotechnology, Dallas, TX, USA; rabbit anti-pHH3 [1:100]; Cell Signaling, Whitby, Ontario, Canada) diluted in sterile PBS at 4 °C. Using a hydrophobic barrier, one section per slide served as an omission control and was incubated in sterile PBS only. Slides were then rinsed with three changes of PBS (2 min each) and sections were subsequently incubated with secondary antibody (Cy3 labelled goat anti-rabbit; PCNA [1:200]; pHH3 [1:250]; Jackson ImmunoResearch Laboratories, West Grove PA, USA) in sterile PBS for one hour at room temperature. Slides were then washed with PBS three times (2 min each) prior to counterstaining with DAPI (4′,6-diamidino-2-phenylindole [1:10,000]; Life Technologies, ThermoFisher Scientific, Waltham, MA, USA) in sterile PBS for two minutes at room temperature. Finally, slides were again washed with PBS three times (2 min each) prior to coverslipping with fluorescent mounting medium (Agilent Technologies Inc., Mississauga, ON, Canada).

#### 2.6.2. Modified Immunofluorescence Protocol with Tris Retrieval

To document the expression of HuCD, and to co-label with each of GFAP and NeuN, double immunofluorescence was conducted using a modified protocol. First, slides were rehydrated to dH_2_O (see above) and washed with PBS for 15 min. Slides were then immersed in a tris base solution (50 mM tris buffered saline with 0.05% Tween 20) for 30 min at 95 °C followed by a 30-min cool at room temperature for antigen retrieval. Slides were then washed in PBS with 0.1% Tween 20 (PBST) for 10 min, followed by two washes of PBS for five minutes each. Sections were then blocked in 10% NGS in sterile PBS with 0.3% Triton-X-100 (Sigma-Aldrich, Oakville, ON, Canada) for 30 min at room temperature before being tipped off and incubated with primary antibody (mouse anti-HuCD [1:10] Molecular Probes, Rockford, IL, USA; rabbit anti-GFAP [1:400] Agilent Technologies Inc., Mississauga, ON, Canada; rabbit anti-NeuN [1:500] Abcam, Cambridge, MA, USA) overnight at 4 °C. Primary antibodies were applied as a cocktail, and diluted in sterile PBS with 1% bovine serum albumin (BSA; Santa Cruz BioTechnology, Santa Cruz, CA, USA). One section per slide was incubated in sterile PBS only as an omission control. Slides were then washed three times with PBS with 1% BSA for 10 min each, followed by the application of secondary antibody (Alexa Fluor-488 labelled goat anti-mouse [Life Technologies, ThermoFisher Scientific, Waltham, MA, USA] for HuCD [1:500]; or Cy3 labelled goat anti-rabbit for GFAP [1:1000] and NeuN [1:1000]. Secondary antibodies were applied as a cocktail and were diluted in sterile PBS for one hour at room temperature. Slides were then washed in PBS three times (2 min each), counterstained with DAPI [1:10,000] in sterile PBS for 5 min at room temperature, and were washed a final time in three changes of PBS (2 min each) prior to coverslipping with fluorescent mounting medium.

#### 2.6.3. Modified Immunofluorescence Protocol with Trypsin Retrieval

To detect each combination of SOX2/BrdU, SOX2/Vimentin, and GFAP/Vimentin, a modified immunofluorescence protocol with two antigen retrieval steps was employed. Slides were rehydrated to dH_2_O (see above) and washed in PBS for 5 min. Slides then underwent antigen retrieval by immersion in citrate buffer (12 min at 95 °C followed by a 20-min cool at room temperature). Slides were then washed again with PBS (2 min) before being incubated in 0.1% trypsin (Sigma-Aldrich, St. Louis, MI, USA), in sterile PBS, for 20 min at 37 °C for further antigen retrieval. After an additional PBS wash (2 min), sections were then blocked in 5% NGS in diluent (1% BSA, 0.5% Tween 20 (Sigma-Aldrich, Oakville, ON, Canada), 0.1% sodium azide [Fisher Scientific, Waltham, MA, USA] in PBS) for 30 min at 37 °C. Next, the blocking solution was tipped off and sections were incubated in primary antibody in diluent overnight at 4 °C (rabbit anti-SOX2 [1:50] Cell Signaling, Whitby, ON, Canada; mouse anti-Vimentin [1:50] Developmental Studies Hybridoma Bank, Iowa City, IA, USA; rabbit anti-GFAP [1:400] Agilent Technologies, Mississauga, ON, Canada; mouse anti-BrdU [1:50] Developmental Studies Hybridoma Bank, Iowa City, IA, USA). One section on each slide served as an omission control and was incubated in diluent without primary antibody. After being washed three times in PBS (2 min each), sections were incubated in secondary antibody (Alexa Fluor-488 labelled goat anti-mouse for Vimentin [1:200] and BrdU [1:200]; or Cy3 labelled goat anti-rabbit for SOX2 [1:200] and GFAP [1:1000] diluted in sterile PBS for one hour at room temperature. Slides were again washed three times in PBS (2 min each) before counterstaining with DAPI [1:10,000] in PBS for 2 min. Slides were washed a final three times in PBS (2 min each) before coverslipping with fluorescent mounting medium.

### 2.7. Quantification of Slow Cycling and Proliferation Data

We used two different strategies to investigate whether: (1) the proportion of proliferating ependymal cells differed along the length of the trunk spinal cord; and (2) the proportion of proliferating ependymal cells was altered in response to tail loss. First, we used our BrdU data generated during the short-duration BrdU experiment. Second, we used a separate experiment with different time points to immunostain for the S phase marker PCNA. Our PCNA data were derived from the trunk spinal cords of original tailed (*n* = 3) and tail regenerating individuals at three different time points: 2 days post-tail loss (*n* = 3); 8 days post-tail loss (*n* = 3); and 12 days post-tail loss (*n* = 3). To reveal any regional differences in the proportion of slow cycling cells (as obtained from the long-duration BrdU pulse-chase experiment) and constitutively proliferating cells, as well as any differences in response to tail loss, we quantified the number of immunopositive ependymal cells in each of the cranial, middle and caudal segments (Figure 1A).

After segmentation, trunk spinal cord segments were sectioned in the transverse plane. Three random sections per trunk spinal cord segment were selected (500 to 2500µm apart) and immunostained with either BrdU or PCNA (Figure 1B). For each section, the ependymal layer was imaged at 40× objective using an Axio Imager D1 Microscope (Carl Zeiss Canada Ltd., Toronto, ON, Canada). Using ImageJ, a 16 µm diameter circle was drawn around the ependymal layer using the Selection Brush tool, originating from the center of the central canal, to circumscribe the region of interest (Figure 1C). The number of ependymal cells (including those immunopositive and immunonegative for the antigen of interest) within or contacting the circle defining the region of interest were manually counted using the Multi-Point tool and were recorded for statistical analysis (Figure 1D). All cell counts were conducted by a counter blinded to the treatment conditions.

### 2.8. Statistical Analyses

All proportions are reported as the mean with a 95% confidence interval. When the probability (*p*) value was less than 0.05 (*p* < 0.05), results were considered statistically significant. All analyses were performed using the software SAS 9.3 (SAS Institute Inc., Cary, NC, USA). To test for differences in the proportion of immuno-positive ependymal cells between groups, a general linear mixed model that included the random effect of lizard and the fixed effects of group (original or tail loss), segment (cranial, middle, caudal), and chase (where applicable; long-duration BrdU experiment, 0 days, 140 days), as well as their interaction, was used. The random effect was coded as: lizard (time [days post-autotomy]) for the PCNA data; lizard (group) for the short-duration BrdU experiment; and lizard (chase) for the long-duration BrdU pulse-chase experiment. Subsamples were averaged within segments, and data were tested for normality with a Shapiro–Wilk test and examination of the residuals; all datasets were normally distributed. A log transformation was used if it improved the distribution, and analyses were performed with a bias correction term of 0.005 (all zeros within the data set were included as 0.005).

## 3. Results

### 3.1. Spinal Cord Structure and Histology

**Figure 2:**
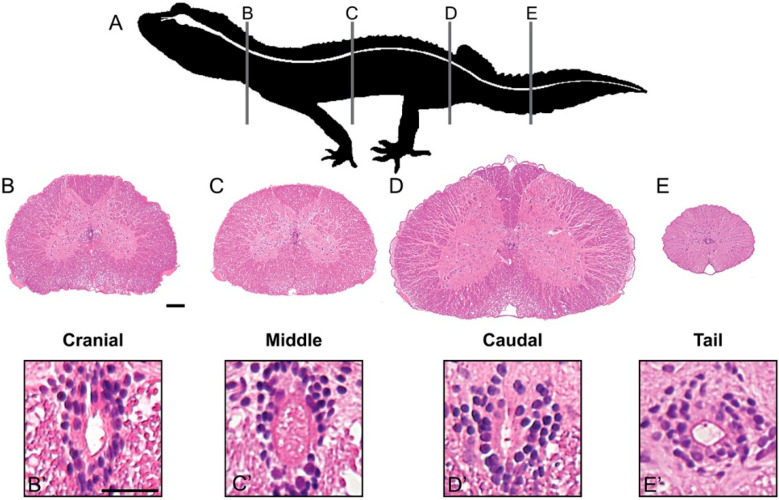
The gecko spinal cord has a consistent histological organization across its length. (**A**) Schematic representation of the leopard gecko (*Eublepharis macularius*) in lateral view. The central nervous system is highlighted in white. Vertical lines indicate the location and plane of section of panels (**B**–**E**). (**B**–**E**) Serial sections of the spinal cord in transverse plane, stained with hematoxylin and Eosin. Note that though the organization remains consistent along the length of the spinal cord, the proportions of grey to white matter and spinal cord diameter vary slightly with position. (**B’**–**E’**) The central canal is lined by a pseudostratified layer of ependymal cells. The central canal and ependymal layer exhibit a more ovoid shape cranially, and progressively round caudally. Note the presence of cilia in the lumen of the central canal. Scale bars: (**B**–**E**) = 100 µm; (**B’**–**E’**) = 25 µm.

**Figure 3:**
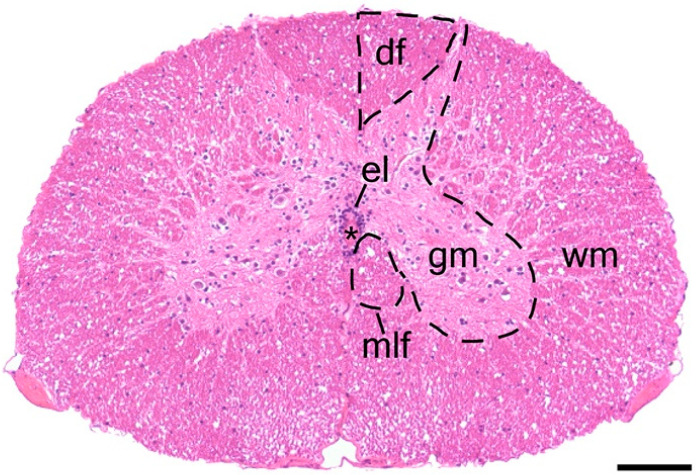
Histology of the gecko trunk spinal cord. A transverse section of the trunk spinal cord stained with hematoxylin and eosin. The spinal cord shows a butterfly-shaped grey matter surrounded by columns of white matter. The dorsal horns of the grey matter divide the white matter into paired dorsal funiculi. The ependymal layer is centrally located in the spinal cord and lines the central canal. Ventral and medial to the ependymal layer are the paired medial longitudinal fasciculi. * = central canal, df = dorsal funiculus, el = ependymal layer, gm = grey matter, mlf = medial longitudinal fasciculus, wm = white matter. Scale bar = 100 µm.

In cross section, the spinal cord is organized around a near-centrally positioned central canal, surrounded by a tubular arrangement of epithelial-like ependymal cells (Figure 2 and Figure 3). Closer inspection reveals that ependymal cells have a pseudostratified arrangement and appear to be ciliated. The central canal is continuous with the ventricular system of the brain and demonstrates a subtle change in shape along the length of the spinal cord, which is mirrored by the ependymal layer (Figure 2B’–E’). Although it is typically ovoid (long axis in the vertical plane) cranially, it becomes circular in shape in more caudal positions.

The ependymal layer is invested within the grey matter (Figure 2 and Figure 3). The grey matter is rich in neuronal soma and is organized into four conspicuous horns, paired dorsally and ventrally; there are no lateral horns. Grey matter is surrounded by white matter, composed mainly of nerve tracts (Figure 3). Ventral to the ependymal layer, nested medially adjacent to the grey matter, are bundles of large, myelinated axons known as the medial longitudinal fasciculus [45]. The boundary between white and grey matter is less distinct than that observed in mammals, but still identifiable. Within the transverse plane, the white matter can be subdivided into right and left sides by dorsal and ventral medial septa. That portion of the white matter between the dorsal septum and the dorsal horns is known as the dorsal funiculus; lateral and ventral funiculi are continuous with each other. The white matter is bounded by the pia mater.

### 3.2. Ependymal Cells of the Trunk Spinal Cord Are a Heterogeneous Population That Includes NSPCs

Across vertebrates, including mammals, resident populations of NSPCs have been identified within the ependymal layer of the trunk spinal cord (e.g., [4,32,46]; see also [24]). In addition, cells expressing various NSPC markers have also been identified in the spinal cord of the gecko tail [10]. To test whether cells from the trunk spinal cord also demonstrated NPSC-like characteristics, and to determine whether they altered their protein expression in response to spinal cord injury (in this case tail loss), we performed immunostaining at three key time points: (1) prior to tail loss (i.e., spinal cord intact); (2) two days post-tail loss (tail spinal cord ruptured); and (3) eight days post-tail loss (tail spinal cord regenerating). As tail autotomy results in the complete rupture of the spinal cord, we exploited this phenomenon to investigate whether a distal spinal cord injury affects ependymal cell marker expression in the trunk spinal cord. We reasoned that the spinal cord is a closed system [47], and hence injuries in one region may elicit an organ-wide response. As an additional comparison, we also sampled spinal cords from original tails. For this analysis, we focused on three classic markers of radial glia, the transcription factor SOX2, and the intermediate filaments Vimentin and glial fibrillary acidic protein (GFAP).

**Figure 4:**
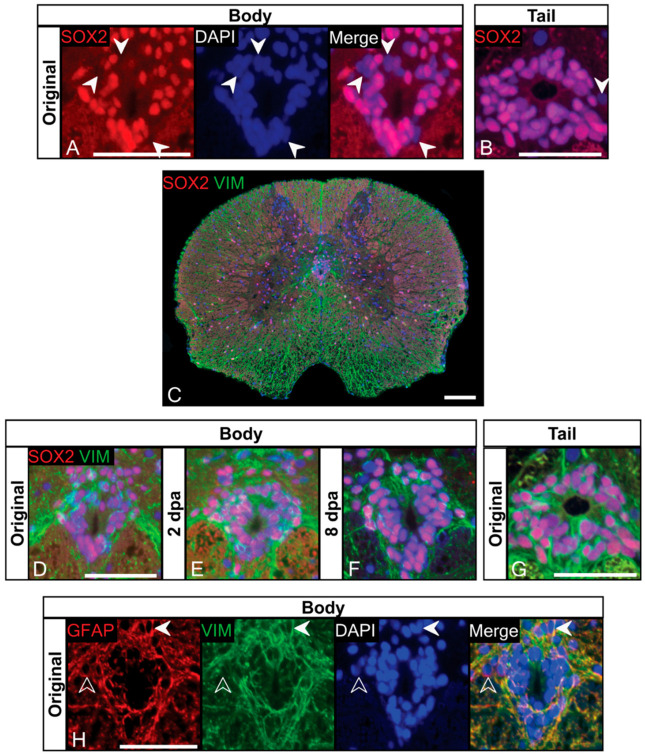
Ependymal cells of the trunk spinal cord express NSPC markers SOX2, Vimentin and GFAP. (**A**,**B**) The majority of ependymal cells in the trunk and tail spinal cord express NSPC marker SOX2. SOX2– ependymal cells are marked with white arrows. (**C**) SOX2/Vimentin expression in the trunk spinal cord. Vimentin expression is largely confined to the ependymal layer and white matter. A fascicle of Vimentin+ fibers is seen projecting toward the pia within the dorsal medial septum. (**D**–**F**) SOX2/Vimentin expression in the ependymal layer of the trunk spinal cord before (**D**), 2 days (**E**) and 8 days (**F**) post-autotomy. Note that expression remains unchanged across time points. (**G**) SOX2/Vimentin is expressed by ependymal cells of the original tail spinal cord, and mirrors expression in the trunk spinal cord. (**H**) A representative section from the cranial segment of the trunk spinal cord. The majority of Vimentin+ ependymal cells co-express radial glia marker GFAP (filled arrows). GFAP+/Vimentin– ependymal cells are also present within the ependymal, slightly offset from the central canal (open arrows). dpa = days post-autotomy. Scale bars: (**A**,**B**,**D**–**F**,**G**,**H**) = 10 µm; (**C**) = 20 µm.

Using immunofluorescence, we determined that most (but not all) ependymal cells along the length of the trunk spinal cord express SOX2 prior to and following tail loss (Figure 4). A similar pattern of SOX2 expression by ependymal cells was also observed in the original tail spinal cord (Figure 4B).

Next, we sought to determine if SOX2+ ependymal cells also expressed Vimentin. Our immunostaining revealed that Vimentin is robustly expressed by ependymal cells along the length of the gecko trunk spinal cord (Figure 4C and Figure S1), including the majority of those immunostaining for SOX2 (Figure 4D). Vimentin expression was particularly obvious as a consolidation of radial processes forming fascicles passing within the dorsal medial septum and ventrolaterally within the ventral horns of the grey matter (Figure 4C). Vimentin+ processes were also found throughout the white matter, radiating toward the pial surface (Figure 4C). Following tail loss (Figure 4E,F), we continued to observe robust co-expression of SOX2 and Vimentin by ependymal cells, with no qualitative differences in immunoreactivity for either marker. Matching the immunostaining pattern observed in the trunk, ependymal cells of the original tail spinal cord were also SOX2+/Vimentin+ (Figure 4G).

**Figure 5:**
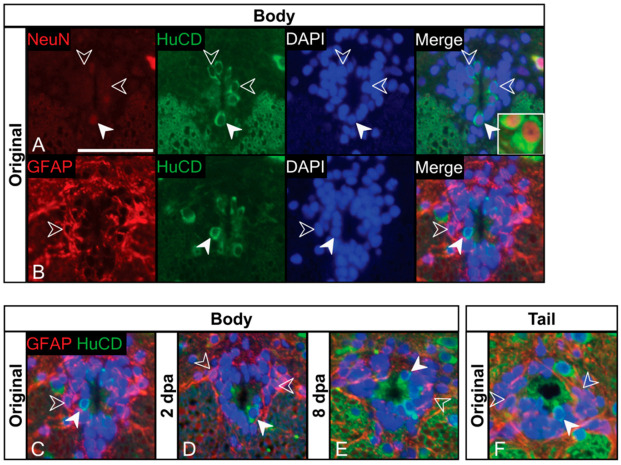
Trunk spinal cord ependymal cells represent a heterogeneous population with distinct GFAP+ and HuCD+ populations. (**A**) HuCD+ cells are present within the trunk spinal cord and are in close contact with the central canal. HuCD+ cells have either weak (filled arrow) or absent NeuN expression (open arrows). Note that NeuN expression in HuCD+/NeuN+ cells does not reach the intensity of NeuN expressing neurons of the grey matter (inset). (**B**) The ependymal layer of the trunk spinal cord contains distinct populations of GFAP+/HuCD–cells (open arrows) and GFAP–/HuCD+ cells (filled arrows). GFAP+ cells reside peripherally in the ependymal layer. (**C**–**E**) GFAP/HuCD expression in the ependymal layer of the trunk spinal cord before (**C**), 2 days (**D**) and 8 days (**E**) post-autotomy. Note that expression remains relatively constant across time points. (**F**) The original tail spinal cord contains distinct GFAP+/HuCD– and GFAP–/HuCD+ populations of ependymal cells. The locations at which these cells reside in the tail mirror what is found in the trunk spinal cord. Representative sections from cranial trunk spinal cord was used for all images. dpa = days post-autotomy. Scale bar: (**A**–**F**) = 10 µm.

Robust expression of SOX2 and Vimentin by ependymal cells points towards their identification as radial glia. To expand our characterization, we then immunostained for GFAP. We found that for most ependymal cells, GFAP co-localized with Vimentin, although rare GFAP+/Vimentin– ependymal cells were also observed (Figure 4H). Characteristically, these cells appeared to be set back from (i.e., abluminal, not in direct contact with) the central canal. Beyond the ependymal layer, GFAP/Vimentin expression was widespread throughout the trunk spinal cord (Figure S2A). In addition, GFAP+ stellate-shaped astrocytes were found at the borders of the ventral horns and adjacent white matter, as well as radially oriented processes throughout the grey and white matter (Figure S2B). Similar to SOX2 and Vimentin, we did not detect any qualitative changes in GFAP expression at any time point following tail loss (Figure 5D,E and Figure S3).

We then asked if any neuronal-like cells contributed to the ependymal layer. We observed large numbers of HuCD+ cells within the ependymal layer, typically in close contact with the lumen of the central canal (Figure 5A). In addition, we also found that some HuCD+ ependymal cells also weakly expressed NeuN. It is worth noting that the weak but detectable NeuN expression in some ependymal cells stands in stark contrast with the robust NeuN (and HuCD) labelling observed in large neurons of the adjacent grey matter (Figure 5A, inset).

To determine the relationship between GFAP and HuCD expressing cells, we next performed double-labelling. We found that these proteins mark two distinct populations: HuCD+/GFAP– cells, which typically contact the central canal; and HuCD–/GFAP+ cells, which occupy abluminal positions (Figure 5B). An additional population of ependymal cells appears to be HuCD–/GFAP–. We observed the same three cell populations in the original tail (Figure 5F). Though not quantified, tail loss did not result in any detectable qualitative change in HuCD or GFAP immunostaining in the trunk spinal cord (Figure 5C–E and Figure S3).

### 3.3. Label-Retaining Cells Are Present in the Ependymal Layer

**Figure 6:**
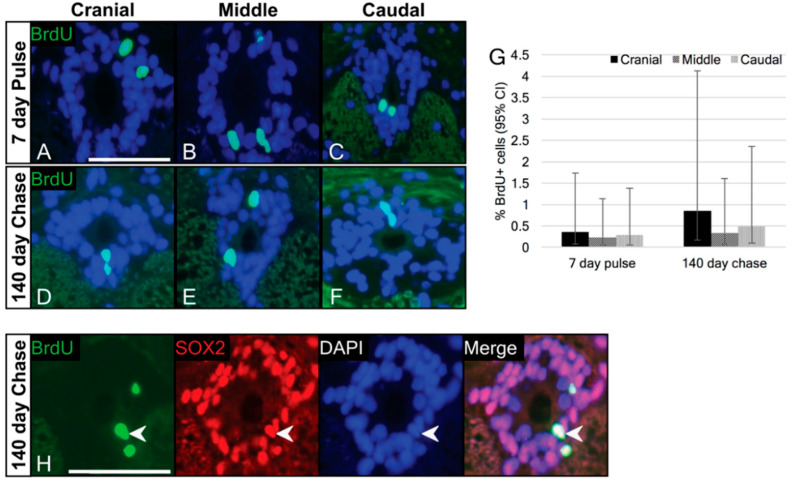
A subset of slow-cycling cells is present within the ependymal layer of the trunk. (**A**–**C**) After 7 days of pulse, BrdU+ cells are present in the ependymal layer of each the cranial (**A**), middle (**B**) and caudal (**C**) segments of spinal cord. (**D**–**F**) After 140 days of chase, BrdU+ cells are still present within the ependymal layer across the trunk spinal cord. (**G**) Quantification of BrdU+ cells in each segment after the pulse and 140-day chase time points. There are no significant regional differences in staining at the pulse or after the chase, nor are there significant differences between the pulse and 140-day chase. (**H**) A representative section from the cranial segment of the trunk spinal cord. BrdU co-localized with SOX2 at the 140-day chase time point (arrows). CI = confidence interval. Scale bars: (**A**–**F**,**H**) = 10 µm.

To determine whether ependymal cells of the trunk spinal cord were quiescent, we conducted a long-duration (20-week) BrdU pulse-chase experiment. At the end of the pulse (0-day chase), BrdU+ ependymal cells were observed in each segment of the trunk spinal cord (Figure 6A–C). BrdU incorporation occurred at proportions of approximately 0.29% in each the cranial, middle and caudal segments (Figure 6G; see Table 2 for proportions and 95% confidence intervals), indicating that a subpopulation of ependymal cells did cycle during the seven-day pulse period. Next, we examined trunk spinal cord tissue at a 140-day chase time point. After 20 weeks, BrdU+ cells were still detectable in the ependymal layer of each of the cranial, middle and caudal segments of the trunk spinal cord (Figure 6D–F) at proportions of approximately 0.55% (Figure 6G; Table 2). To determine if there were any significant differences between the pulse (0 days) and chase (140 days) groups, we conducted a statistical analysis using the general linear mixed model. This analysis revealed that there were no statistically significant differences in regional variation at the pulse or end of the chase (*p* values > 0.05), nor was there a statistically significant loss of the label following the chase (*p* = 0.4776; Figure 6G). These findings suggest that there is no dilution of the label taking place, and that the majority of cells incorporating the BrdU label were slow-cycling/quiescent.

Finally, we sought to determine if the label-retaining cells (BrdU+, 140-day chase) of the ependymal layer also express the NSPC marker SOX2. Using double immunofluorescence, we found that after 140 days of chase, BrdU+ cells also express SOX2 (Figure 6H).

### 3.4. Cells of the Ependymal Layer Constitutively Proliferate

**Figure 7:**
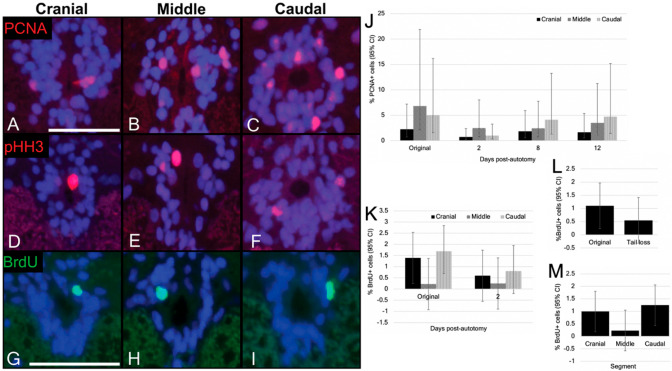
Ependymal cells of the trunk spinal cord constitutively proliferate. (**A**–**I**) In original tailed geckos, PCNA+ (**A**–**C**), pHH3+ (**D**–**F**) and BrdU+ (**G**–**I**) cells are present within the ependymal layer across all three segments of trunk spinal cord. (**J**) Quantification of PCNA+ ependymal cells across the trunk spinal cord before, 2 days, 8 days and 12 days after tail loss. (**K**) Quantification of BrdU+ ependymal cells across the trunk spinal cord after two-day pulse prior to and following tail loss. (**L**) Quantification of BrdU+ ependymal cells in original tail and tail loss groups. Comparing BrdU incorporation between groups reveals no significant difference in the proportion of BrdU+ cells after tail loss (*p* = 0.2785). (**M**) Quantification of BrdU+ cells in each segment of the trunk spinal cord. Combining data across time points reveals there is no segment variation in BrdU staining across the trunk spinal cord (*p* values > 0.05). CI = confidence interval. Scale bars: (**A**–**I**) = 10 µm.

We identified proliferating cells using immunostaining for all three proliferation markers (PCNA, pHH3, and the short-duration BrdU experiment) in each of the three trunk spinal cord segments (Figure 7A–I). Next, we quantified the number of immunopositive cells. Using PCNA, the percentage of immunolabelled ependymal cells was less than 7% in each segment (Figure 7J; Table 3). Although our immunostaining protocol did label pHH3+ cells, the low number of immunostained cells observed (five out of the ~1350 cells counted across 23 sections) rendered a quantitative analysis impractical.

For the short-duration BrdU labelling experiment, we identified BrdU+ cells in the ependymal layer of each segment of trunk spinal cord (Figure 7G–I) at a proportion of approximately 1.10% (Figure 7K; Table 4), indicating that a subset of ependymal cells did undergo cell division during the two-day injection period. Statistical analysis revealed no significant regional differences in positivity across the trunk spinal cord (*p* values > 0.05).

### 3.5. Ependymal Cell Proliferation Is Unaltered in Response to Tail Loss

**Figure 8:**
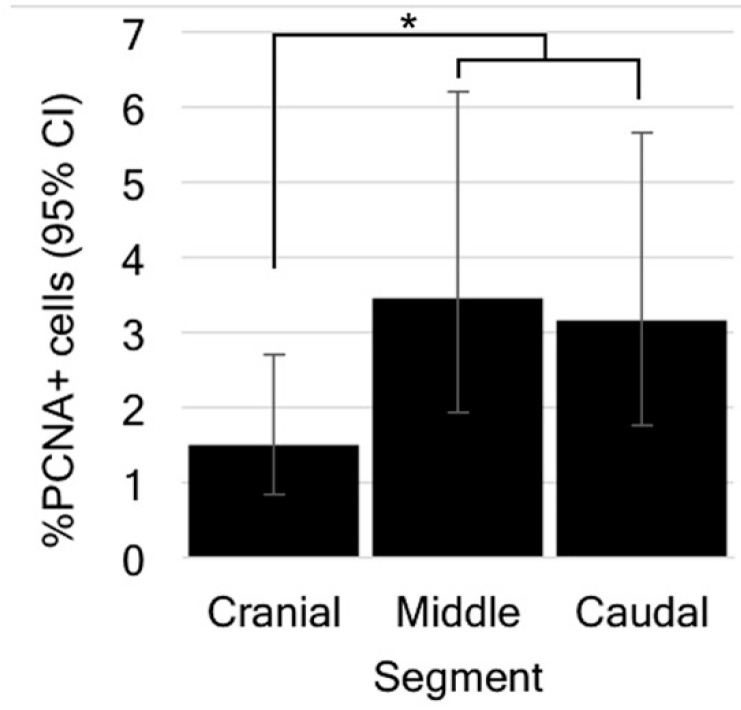
The proportion of PCNA+ cells varies along the length of the trunk spinal cord. Combining data across time points reveals a significantly reduced proportion of PCNA+ cells in the cranial segment of trunk spinal cord compared to the middle (*p* = 0.0179) and caudal (*p* = 0.0321) segments. CI = confidence interval. Asterisk (*) indicates significance (*p* < 0.05).

We were then interested in determining if ependymal cell proliferation was altered in response to spinal cord injury. Given the low number of pHH3+ cells observed in our constitutive proliferation experiment, we limited our investigation to PCNA and the short-duration BrdU labelling protocol. PCNA immunostaining was conducted on trunk spinal cords 2 days, 8 days, and 12 days post-tail loss. While PCNA+ cells were detected in the ependymal layer of each segment at each time point post-tail loss, our quantitative analysis revealed that the proportion of ependymal cells expressing this marker was less than 5% for each segment (Figure 7J; Table 3). However, given that the interaction between time point and segment was nonsignificant, looking at the effect of segment revealed a significantly higher proportion of cell proliferation in the caudal segment compared to the cranial segment (*p* = 0.0321), and in the middle segment compared to the cranial segment (*p* = 0.0179; Figure 8). These data indicate that ependymal cells of the middle and caudal segments of the trunk spinal cord are more proliferative than those of the cranial segment. Additionally, proportions of PCNA+ cells were not significantly changed across time points (*p* values > 0.05).

Next, we investigated the effect of tail loss on BrdU incorporation. Using our short-duration BrdU labelling approach, geckos were induced to autotomize and then immediately pulsed with BrdU (twice daily i.p. injections, 50 mg/kg) for two days, and were then collected. At the end of the pulse, autotomized geckos did demonstrate BrdU incorporation in the ependymal layer of the cranial, middle and caudal segments (Figure 7K; see Table 4). Statistical analysis revealed a trend of reduced BrdU incorporation in post-autotomy geckos (approximately 0.55%), but this group effect did not reach statistical significance (*p* = 0.2785; Figure 7L). In addition, there were no differences detected between segments (*p* values > 0.05; Figure 7M).

## 4. Discussion

Here, we conducted a spatiotemporal characterization of trunk spinal cord ependymal cells in the leopard gecko. We determined that ependymal cells represent a heterogeneous population. Most (but not all) ependymal cells express SOX2 and, similar to radial glia, many express the intermediate filaments GFAP+ and Vimentin (but do not co-express the neuronal marker HuCD). A second population of ependymal cells, herein identified as CSF-c cells, express HuCD (and sometimes NeuN), but not GFAP. To study cell dynamics, we used a long-duration BrdU experiment and determined that the majority of mitotically active cells are slow-cycling. Next, we quantified the S phase marker PCNA and, in a separate experiment, BrdU following a short-duration labelling experiment. Both assays revealed that cells of the ependymal layer do actively cycle under homeostatic conditions. Interestingly, our PCNA data and short-duration BrdU experiment revealed that this proliferation is unaltered in response to the distal spinal cord injury produced by tail loss. Taken together, our data suggest that, like the tail, the trunk spinal cord of geckos includes populations of radial glia within the ependymal layer.

Consistent with their identification as NSPCs, we found that the majority of ependymal cells expressed a panel of protein markers characteristic of radial glia, including the intermediate filaments GFAP and Vimentin, and the transcription factor SOX2. Whereas SOX2 expression by ependymal cells is often limited prior to injury, even among regeneration-competent species [32,46,48,49], we observed widespread immunostaining for this transcription factor in geckos before their tails were removed (see also [11]). Similarly, near ubiquitous expression of SOX2 has also been documented in the spinal cord of the tail [10,11], which does regenerate, as well as in the mammalian spinal cord, which does not [43,50,51]. By way of explanation, it has been suggested that levels of intracellular SOX2, and not its ubiquity among the ependymal population, is the key to promoting neural tissue restoration [34,52,53]. For example, neural progenitor cell defects are observed when SOX2 levels drop below 20%–30% of wildtype levels [54,55,56], while overexpression of SOX2 inhibits neurogenesis and promotes gliogenesis in neurosphere cultures [52].

Along with SOX2, ependymal cells of the trunk spinal cord also co-label with Vimentin. Vimentin is one of the first intermediate filaments to be expressed by embryonic neuroprogenitor cells, known as neuroepithelial cells [57,58,59]. Although Vimentin is commonly replaced by GFAP during development [35], it is retained by many–but not all–species capable of neuroregeneration [8,36,60,61]. For example, among tail-regenerating lizards, Vimentin expression within the spinal cord has been reported for *Podarcis sicula* but not *Anolis sagrei* [23,62]. Further supporting their identification as radial glia, most Vimentin+ cells co-expressed GFAP. We also observed GFAP+ cells that lacked Vimentin. One explanation is that these cells may reflect a more mature subset of radial glia-like cells, having completed the ontogenetic shift from Vimentin to GFAP.

In addition to expressing various proteins associated with NSPCs, we also determined that ependymal cells of the trunk spinal cord include populations that are quiescent. Quiescence, or slow-cycling, is commonly reported for stem/progenitor cell populations across various tissues [31,63,64,65,66], including the CNS [12,29,67,68]. As currently understood, quiescence is a mechanism to avoid senescence and preserve the progenitor pool for unforeseen circumstances, such as injury [53]. Using a long-duration (20-week) BrdU pulse-chase paradigm, we confirmed that ependymal cells of the trunk spinal cord are label-retaining. Significantly, at 20 weeks all BrdU+ cells also expressed SOX2. While we did not determine the identity of the BrdU+ cells, previous studies have confirmed that radial glia, including those from the spinal cord, are typically slow-cycling [12,27,69]. Given that the proportion of label-retaining cells in the gecko spinal cord was not reduced between the pulse (7 days) and chase (140 days), we predict that the majority of ependymal cells are slow cycling. Our data suggest that any cells that cycle and acquire the label during the pulse then go on to retain it for relatively long periods of time, up to 140 days. The limited population labelled with BrdU at 140 days of chase is likely reflective of the short length of the pulse, and not the size of the slow-cycling population. Although our long-duration BrdU data clearly demonstrate that a largely quiescent population resides within the spinal cord, whether other cells within the ependymal pool are capable of more rapid constitutive proliferation remains unclear. Among other species, including neurogenic regions of the zebrafish [29] and mouse [67,68] brain, actively proliferating and quiescent stem/progenitor cells are known to coexist.

We documented ependymal cells in both the S phase (PCNA) and M phase (pHH3) of the cell cycle in each of the three trunk spinal cord segments. The differences observed in the proportion of pHH3+ vs. PCNA+ cells (pHH3 expressing cells were far less common than cells immunostaining for PCNA) is best explained by the difference in the relative duration of each phase [38,70]. However, it should be noted that, since both PCNA and BrdU are proxies for the S phase of the cell cycle [38,71], we might expect that proportions of PCNA+ cells (~4.7%) and BrdU+ cells after the two-day pulse (~1.1%) be more comparable. Similar quantitative differences between PCNA and BrdU have been reported elsewhere (e.g., various tissues in canines; [72]). By way of explanation, it has been noted that PCNA immunostaining also labels cells undergoing DNA repair [72]. While BrdU is also incorporated during DNA repair processes, it has been suggested that the concentrations commonly used are insufficient to detect DNA repair [73,74].

Another important finding was that the trunk spinal cord demonstrates regional variation with respect to the proportion of PCNA+ cells. More specifically, cell proliferation was significantly less abundant among the cranial-most populations of ependymal cells. Although a comparable pattern of proliferative polarity (most abundant in the caudal-most populations) of spinal cord cells has also been reported for brown knifefish [75], data from mammals remain equivocal [43,76].

The ability to voluntarily detach (autotomize) the tail is an important adaptation for many species of lizard, including the gecko. Tail autotomy results in the complete rupture of the spinal cord and represents a traumatic and yet readily survivable injury. Unexpectedly, our BrdU labelling strategy found that ependymal cell proliferation was not significantly altered following tail loss. Reduction in proliferation cranial to the site of spinal cord injury has been documented in the rat, where a cervical spinal cord injury resulted in reduced BrdU incorporation in both the olfactory bulb and dentate gyrus of the hippocampus [47]. Paradoxically, in teleost fish, transectional spinal cord injuries increase cell proliferation outside the area of injury. For example, in zebrafish, cell proliferation increases within the ependymal layer both cranial and caudal to the lesion site [12]. Furthermore, in the black ghost knifefish, a high-level transectional spinal cord injury increased cell proliferation at locations distant from the injury, with the largest increase observed at the tip of the tail [75]. An important caveat, however, is that the knifefish study did not focus exclusively on the ependymal layer. Interestingly, after contusion injury at the thoracic level in mice, a wave of ependymal proliferation was observed cranially along the spinal cord but was not detected caudal to the lesion site [77]. Combined, these data suggest that the CNS is a closed system, in which injuries to one region produce a system-wide response. Furthermore, the response of the spinal cord at sites distant to the site of injury in the abovementioned species, regardless of regenerative competence, is an increase in proliferation. Similarly, limb amputation in axolotls has recently been shown to increase cell cycle activation and proliferation in intact, uninjured tissues across the body, including the contralateral limb, heart, liver and ependymal layer of the spinal cord [78]. We speculate that the failure of the gecko spinal cord to become activated in the same way represents an adaption associated with the evolution of tail autotomy. However, we predict that they likely would become activated in response to a direct, trunk spinal cord injury given that their phenotype so closely resembles the phenotype of NSPCs within the regeneration-competent tail spinal cord. Whether this response would result in functional recovery or glial scarring remains to be seen.

In addition to radial glia, we also detected cells with a neuronal phenotype, contributing to the complexity of the ependymal population. More specifically, we identified populations of cells immunostaining for HuCD (and sometimes HuCD and NeuN). Importantly, HuCD+ cells were GFAP–. We recognize these cells as cerebrospinal fluid-contacting (CSF-c) cells (= central canal-contacting cells, liquor-contacting neurons, or cerebrospinal fluid-contacting neurons; [39,40,42,79]). Similar populations of intermediate filament-bearing radial glia, representing NSPCs, and HuCD-expressing CSF-c cells have been documented in the gecko tail [10]. CSF-c cells have been identified in many vertebrate species, from zebrafish to primates [39,41,42,44,80,81,82], as well as several species of lizard [83,84,85,86,87]. In our investigation of geckos, CSF-c cells were located immediately adjacent to the lumen of the central canal, with a small, bulb-like apical process. Although NeuN expression is not typically associated with CSF-c cells [40,42,43,44], weak expression has been observed in the rat [81]. Despite expressing SOX2, CSF-c cells (unlike radial glia) are not considered to represent an NSPC population. Instead, they appear to function as mechanosensory cells or receptors that monitor the pH of cerebrospinal fluid [40,41,42,79,84,85,86,87]. In other lizard species, CSF-c cells have both cilia and stereocilia [18,83,85,87]), are gamma-aminobutyric acid (GABA)-ergic [86,87] and produce exosomes [87]. CSF-c cells have also been identified in the trunk spinal cord following a transectional injury [reviewed in 87].

Among amniotes, lizards are unique in being able to spontaneously regenerate the spinal cord following a complete amputation (autotomy). Our findings demonstrate that—similar to the regeneration-competent tail—the entire length of the trunk spinal cord contains populations of constitutively proliferative NSPC-like cells. We predict that the same regenerative capacity that is provided to the tail spinal cord is also maintained in the trunk spinal cord. Given the parallels between the tail and trunk spinal cords, we propose that the gecko tail spinal cord is a relevant and humane model for the study of transectional-type spinal cord injuries and gap replacement investigations (sensu [9]).

## Figures and Tables

**Table 1:** Summary table of optimized immunofluorescent protocols for proteins of interest.

Antigen	Retrieval	Block	Primary	Secondary
BrdU (Anti-5-bromo-2′-deoxyuridine)	Citrate buffer 12 min at 95 °C, 20 min at room temperature in solution 0.1% trypsin in PBS 20 min at 37 °C	5% NGS in diluent 30 min at 37 °C	1:100 in diluent overnight at 4 °C (DSHB, mouse monoclonal, G3G4, RRID: AB_2618097)	1:200 in sterile 1XPBS for 1 h at room temperature (Goat anti-Mouse AlexaFluor 488, Life Technologies, A11001, RRID: AB_2534069)
GFAP (Anti-glial fibrillary acidic protein)	Citrate buffer 12 min at 95 °C, 20 min at room temperature in solution 0.1% trypsin in PBS 20 min at 37 °C	5% NGS in diluent 30 min at 37 °C	1:400 in diluent overnight at 4 °C (DAKO, rabbit polyclonal, Z0334, RRID: AB_10013382)	1:1000 in 1XPBS for 1 h at room temperature (Cy3-conjugated Goat anti-Rabbit IgG, 111-165-144, RRID: AB_2338006)
HuCD (Anti-human neuronal protein HuC/HuD)	Tris buffer 30 min at 95 °C, 30 min at room temperature in solution	10% NGS in 0.3% Triton-X-100 in 1XPBS 30 min at room temperature	1:10 in 1%BSA in 1XPBS overnight at 4 °C (Molecular Probes, mouse monoclonal, 16A11, RRID: AB_221448)	1:500 in sterile 1XPBS for 1 h at room temperature (Goat anti-Mouse AlexaFluor 488, Life Technologies, A11001, RRID: AB_2534069)
NeuN (Anti-neuronal nuclei)	Tris buffer 30 min at 95 °C, 30 min at room temperature in solution	10% NGS in 0.3% Triton-X-100 in 1XPBS 30 min at room temperature	1:500 in 1%BSA in 1XPBS overnight at 4 °C (Abcam, rabbit polyclonal, ab104225, RRID: AB_10711153)	1:1000 in 1XPBS for 1 h at room temperature (Cy3-conjugated Goat anti-Rabbit IgG, 111-165-144, RRID: AB_2338006)
PCNA (Anti-proliferating cell nuclear antigen)	none	3% NGS in 1XPBS 1 h at room temperature	1:100 in1XPBS overnight at 4 °C (Santa Cruz Biotech., rabbit polyclonal, sc-7907, RRID: AB_2160375)	1:200 in 1XPBS for 1 h at room temperature (Cy3-conjugated Goat anti-Rabbit IgG, 111-165-144, RRID: AB_2338006)
pHH3 (Anti-phospho-histone H3 (Ser10))	Citrate buffer 12 min at 95 °C, 20 min at room temperature in solution	3% NGS in 1XPBS 1 h at room temperature	1:100 in1XPBS overnight at 4 °C (Cell Signaling, rabbit polyclonal, 3377S, RRID: AB_1549592)	1:250 in 1XPBS for 1 h at room temperature (Cy3-conjugated Goat anti-Rabbit IgG, 111-165-144, RRID: AB_2338006)
SOX2 (Anti-SRY (sex-determining box region Y) box2)	Citrate buffer 12 min at 95 °C, 20 min at room temperature in solution 0.1% trypsin in PBS 20 min at 37 °C	5% NGS in diluent 30 min at 37 °C	1:50 in diluent overnight at 4 °C (Cell Signaling, rabbit polyclonal, 2748S, RRID: AB_823640)	1:200 in 1XPBS for 1 h at room temperature (Cy3-conjugated Goat anti-Rabbit IgG, 111-165-144, RRID: AB_2338006)
Vimentin (Anti-Vimentin)	Citrate buffer 12 min at 95 °C, 20 min at room temperature in solution 0.1% trypsin in PBS 20 min at 37 °C	5% NGS in diluent 30 min at 37 °C	1:50 in diluent overnight at 4 °C (DSHB, mouse monoclonal, H5, RRID: AB_528506)	1:200 in sterile 1XPBS for 1 h at room temperature (Goat anti-Mouse AlexaFluor 488, Life Technologies, A11001, RRID: AB_2534069)

BSA, bovine serum albumin; DSHB, Developmental Studies Hybridoma Bank, University of Iowa, USA; NGS, normal goat serum; PBS, phosphate buffered saline; RRID, Research Resource Identifier.

**Table 2:** Proportion of BrdU+ ependymal cells and 95% confidence intervals for the long-duration (7 day pulse, 140 chase) pulse-chase experiment.

Chase (Days)	Segment	Mean (%)	95% Confidence Interval	
			Lower	Upper
0	cranial	0.356	0.073	1.733
	middle	0.233	0.048	1.132
	caudal	0.285	0.058	1.323
140	cranial	0.848	0.174	4.126
	middle	0.330	0.068	1.605
	caudal	0.485	0.100	2.360

BrdU, 5-bromo-2′-deoxyuridine.

**Table 3:** Proportion of PCNA+ ependymal cells and 95% confidence intervals.

Group	Segment	Mean (%)	95% Confidence Interval	
			Lower	Upper
Original	cranial	2.242	0.697	7.210
	middle	6.809	2.118	21.894
	caudal	5.033	1.055	16.183
2 dpa	cranial	0.740	0.230	2.380
	middle	2.496	0.776	8.024
	caudal	1.013	0.315	3.256
8 dpa	cranial	1.838	0.277	5.911
	middle	2.415	0.751	7.765
	caudal	4.125	0.572	13.262
12 dpa	cranial	1.669	0.519	5.368
	middle	3.493	1.086	11.232
	caudal	4.723	1.469	15.185

PCNA, proliferating cell nuclear antigen; dpa, days post-autotomy.

**Table 4:** Proportion of BrdU+ ependymal cells and 95% confidence intervals for the short-duration (2 day pulse, 0 day chase) experiment.

Group	Segment	Mean (%)	95% Confidence Interval	
			Lower	Upper
Original	cranial	1.391	0.244	2.538
	middle	0.218	−0.929	1.365
	caudal	1.691	0.544	2.838
2 dpa	cranial	0.595	−0.552	1.742
	middle	0.247	−0.900	1.394
	caudal	0.800	−0.347	1.947

BrdU, 5-bromo-2′-deoxyuridine; dpa, days post-autotomy.

## Data Availability

The data presented in this study are available within the article and Supplementary Materials.

## Supplementary Materials

The following supporting information can be downloaded at: https://www.mdpi.com/article/10.3390/jdb10020021/s1, Figure S1: SOX2/Vimentin is expressed by the ependymal layer of the trunk spinal cord, and is unchanged after tail loss; Figure S2: Radially-oriented GFAP+ processes and astrocytes are present within the white matter of the trunk spinal cord; Figure S3: GFAP/HuCD is expressed by the ependymal layer of the trunk spinal cord, and is unchanged after tail loss.

## References

[1] McDonald J.W., Sadowsky C. (2002). Spinal-cord injury. Lancet.

[2] Yiu G., He Z. (2006). Glial inhibition of CNS axon regeneration. Nat. Rev. Neurosci..

[3] Karimi-Abdolrezaee S., Billakanti R. (2012). Reactive astrogliosis after spinal cord injury—Beneficial and detrimental effects. Mol. Neurobiol..

[4] Meletis K., Barnabé-Heider F., Carlén M., Evergren E., Tomilin N., Shupliakov O., Frisén J. (2008). Spinal cord injury reveals multilineage differentiation of ependymal cells. PLoS Biol..

[5] Barnabé-Heider F., Göritz C., Sabelström H., Takebayashi H., Pfrieger F.W., Meletis K., Frisén J. (2010). Origin of new glial cells in intact and injured adult spinal cord. Cell Stem Cell..

[6] Mothe A.J., Tator C.H. (2005). Proliferation, migration, and differentiation of endogenous ependymal region stem/progenitor cells following minimal spinal cord injury in the adult rat. Neuroscience.

[7] Barnabé-Heider F., Frisén J. (2008). Stem cells for spinal cord repair. Cell Stem Cell.

[8] Dawley E.M., Samson S.O., Woodard K.T., Matthias K.A. (2012). Spinal cord regeneration in a tail autotomizing urodele. J. Morphol..

[9] Szarek D., Marycz K., Lis A., Zawada Z., Tabakow P., Laska J., Jarmundowicz W. (2015). Lizard tail spinal cord: A new experimental model of spinal cord injury without limb paralysis. FASEB J..

[10] Gilbert E.A.B., Vickaryous M.K. (2018). Neural stem/progenitor cells are activated during tail regeneration in the leopard gecko (*Eublepharis macularius*). J. Comp. Neurol..

[11] Sun A.X., Londono R., Hudall M.L., Tuan R.S., Lozito T.P. (2018). Differences in neural stem identity and differentiation capacity drive divergent regenerative outcomes in lizards and salamanders. Proc. Natl. Acad. Sci. USA.

[12] Reimer M.M., Sörensen I., Kuscha V., Frank R.E., Liu C., Becker C.G., Becker T. (2008). Motor neuron regeneration in adult zebrafish. J. Neurosci..

[13] Monaghan J.R., Walker J.A., Page R.B., Putta S., Beachy C.K., Voss S.R. (2007). Early gene expression during natural spinal cord regeneration in the salamander Ambystoma mexicanum. J. Neurochem..

[14] Zhou Y., Xu Q., Li D., Zhao L., Wang Y., Liu M., Gu X., Liu Y. (2013). Early neurogenesis during caudal spinal cord regeneration in adult *Gekko japonicus*. J. Mol. Histol..

[15] Alibardi L. (2014). Observations on lumbar spinal cord recovery after lesion in lizards indicates regeneration of a cellular and fibrous bridge reconnecting the injured cord. J. Dev. Biol..

[16] Lin J.W., Chen Y.R., Wang Y.H., Hung K.C., Lin S.M. (2017). Tail regeneration after autotomy revives survival: A case from a long-term monitored lizard population under avian predation. Proc. R. Soc. B..

[17] Lozito T.P., Londono R., Sun A.X., Hudnall M.L. (2021). Introducing dorsoventral patterning in adult regenerating lizard tails with gene-edited embryonic neural stem cells. Nat. Commun..

[18] Alibardi L. (1994). H3-thymidine labeled cerebrospinal fluid contacting cells in the regenerating caudal spinal cord of the lizard *Lampropholis*. Ann. Anat..

[19] Jacyniak K., McDonald R.P., Vickaryous M.K. (2017). Tail regeneration and other phenomena of wound healing and tissue restoration in lizards. J. Exp. Biol..

[20] Briona L.K., Dorsky R.I. (2014). Radial glial progenitors repair the zebrafish spinal cord following transection. Exp. Neurol..

[21] Than-Trong E., Bally-Cuif L. (2015). Radial glia and neural progenitors in the adult zebrafish central nervous system. Glia.

[22] Johnson K., Barragan J., Bashiruddin S., Smith C.J., Tyrrell C., Parsons M.J., Doris R., Kucenas S., Downes G.B., Velez C.M. (2016). Gfap-positive radial glial cells are an essential progenitor population for later-born neurons and glia in the zebrafish spinal cord. Glia.

[23] Lazzari M., Franceschini V. (2005). Astroglial cells in the central nervous system of the adult brown anole lizard, *Anolis sagrei*, revealed by intermediate filament immunohistochemistry. J. Morphol..

[24] Tanaka E.M., Ferretti P. (2009). Considering the evolution of regeneration in the central nervous system. Nat. Rev. Neurosci..

[25] Becker C.G., Becker T. (2015). Neuronal regeneration from ependymo-radial glial cells: Cook, little pot, cook!. Dev. Cell.

[26] Alvarez-Buylla A., García-Verdugo J.M., Tramontin A.D. (2001). A unified hypothesis on the lineage of neural stem cells. Nat. Rev. Neurosci..

[27] Doetsch F. (2003). The glial identity of neural stem cells. Nat. Neurosci..

[28] Kriegstein A., Alvarez-Buylla A. (2009). The glial nature of embryonic and adult neural stem cells. Annu. Rev. Neurosci..

[29] Grandel H., Kaslin J., Ganz J., Wenzel I., Brand M. (2006). Neural stem cells and neurogenesis in the adult zebrafish brain: Origin, proliferation dynamics, migration and cell fate. Dev. Biol..

[30] Chapouton P., Skupien P., Hesl B., Coolen M., Moore J.C., Madelaine R., Kremmer E., Faus-Kessler T., Blader P., Lawson N.D. (2010). Notch activity levels control the balance between quiescence and recruitment of adult neural stem cells. J. Neurosci..

[31] Fuchs E. (2009). The tortoise and the hair: Slow-cycling cells in the stem cell race. Cell.

[32] Hui S.P., Nag T.C., Ghosh S. (2015). Characterization of proliferating neural progenitors after spinal cord injury in adult zebrafish. PLoS ONE.

[33] Takahashi K., Yamanaka S. (2006). Induction of pluripotent stem cells from mouse embryonic and adult fibroblast cultures by defined factors. Cell.

[34] Graham V., Khudyakov J., Ellis P., Pevny L. (2003). SOX2 functions to maintain neural progenitor identity. Neuron.

[35] Oudega M., Marani E. (1991). Expression of vimentin and glial fibrillary acidic protein in the developing rat spinal cord: An immunocytochemical study of the spinal cord glial system. J. Anat..

[36] Dervan A.G., Roberts B.L. (2003). Reaction of spinal cord central canal cells to cord transection and their contribution to cord regeneration. J. Comp. Neurol..

[37] Tapia C., Kutzner H., Mentzel T., Savic S., Baumhoer D., Glatz K. (2006). Two mitosis-specific antibodies, MPM-2 and phospho-histone H3 (Ser28), allow rapid and precise determination of mitotic activity. Am. J. Surg. Pathol..

[38] Halbach O.V. (2011). Immunohistological markers for proliferative events, gliogenesis, and neurogenesis within the adult hippocampus. Cell Tissue Res..

[39] Roberts B.L., Maslam S., Scholten G., Smit W. (1995). Dopaminergic and GABAergic cerebrospinal fluid-contacting neurons along the central canal of the spinal cord of the eel and trout. J. Comp. Neurol..

[40] Marichal N., García G., Radmilovich M., Trujillo-Cenóz O., Russo R.E. (2009). Enigmatic central canal contacting cells: Immature neurons in “standby mode”?. J. Neurosci..

[41] Djenoune L., Khabou H., Joubert F., Quan F.B., Nunes Figueiredo S., Bodineau L., Del Bene F., Burcklé C., Tostivint H., Wyart C. (2014). Investigation of spinal cerebrospinal fluid-contacting neurons expressing PKD2L1: Evidence for a conserved system from fish to primates. Front. Neuroanat..

[42] Petracca Y.L., Sartoretti M.M., Di Bella D.J., Marin-Burgin A., Carcagno A.L., Schinder A.F., Lanuza G.M. (2016). The late and dual origin of cerebrospinal fluid-contacting neurons in the mouse spinal cord. Development.

[43] Alfaro-Cervello C., Soriano-Navarro M., Mirzadeh Z., Alvarez-Buylla A., Garcia-Verdugo J.M. (2012). Biciliated ependymal cell proliferation contributes to spinal cord growth. J. Comp. Neurol..

[44] Russo R.E., Fernández A., Reali C., Radmilovich M., Trujillo-Cenóz O. (2004). Functional and molecular clues reveal precursor-like cells and immature neurones in the turtle spinal cord. J. Physiol..

[45] Cruce W.L.R., Gans C., Northcutt R.G., Ulinkski P. (1979). Spinal Cord in Lizards. Biology of Reptilia.

[46] Ogai K., Nakatani K., Hisano S., Sugitani K., Koriyama Y., Kato S. (2014). Function of Sox2 in ependymal cells of lesioned spinal cords in adult zebrafish. Neurosci. Res..

[47] Felix M.S., Popa N., Djelloul M., Boucraut J., Gauthier P., Bauer S., Matarazzo V.A. (2012). Alteration of forebrain neurogenesis after cervical spinal cord injury in the adult rat. Front. Neurosci..

[48] Gaete M., Muñoz R., Sánchez N., Tampe R., Moreno M., Contreras E.G., Lee-Liu D., Larraín J. (2012). Spinal cord regeneration in *Xenopus* tadpoles proceeds through activation of Sox2-positive cells. Neural Dev..

[49] Hui S.P., Sengupta D., Lee S.G., Sen T., Kundu S., Mathavan S., Ghosh S. (2014). Genome wide expression profiling during spinal cord regeneration identifies comprehensive cellular responses in zebrafish. PLoS ONE.

[50] Brazel C.Y., Limke T.L., Osborne J.K., Miura T., Cai J., Pevny L., Rao M.S. (2005). Sox2 expression defines a heterogeneous population of neurosphere-forming cells in the adult murine brain. Aging Cell.

[51] Lee H.J., Wu J., Chung J., Wrathall J.R. (2013). SOX2 expression is upregulated in adult spinal cord after contusion injury in both oligodendrocyte lineage and ependymal cells. J. Neurosci. Res..

[52] Bani-Yaghoub M., Tremblay R.G., Lei J.X., Zhang D., Zurakowski B., Sandhu J.K., Smith B., Ribecco-Lutkiewicz M., Kennedy J., Walker P.R. (2006). Role of Sox2 in the development of the mouse neocortex. Dev. Biol..

[53] Hutton S.R., Pevny L.H. (2011). SOX2 expression levels distinguish between neural progenitor populations of the developing dorsal telencephalon. Dev. Biol..

[54] Cavallaro M., Mariani J., Lancini C., Latorre E., Caccia R., Gullo F., Valotta M., DeBiasi S., Spinardi L., Ronchi A. (2008). Impaired generation of mature neurons by neural stem cells from hypomorphic Sox2 mutants. Development.

[55] Ferri A.L., Cavallaro M., Braida D., Di Cristofano A., Canta A., Vezzani A., Ottolenghi S., Pandolfi P.P., Sala M., DeBiasi S. (2004). Sox2 deficiency causes neurodegeneration and impaired neurogenesis in the adult mouse brain. Development.

[56] Taranova O.V., Magness S.T., Fagan B.M., Wu Y., Surzenko N., Hutton S.R., Pevny L.H. (2006). SOX2 is a dose-dependent regulator of retinal neural progenitor competence. Genes Dev..

[57] Schnitzer J., Franke W.W., Schachner M. (1981). Immunocytochemical demonstration of vimentin in astrocytes and ependymal cells of developing and adult mouse nervous system. J. Cell Biol..

[58] Tapscott S.J., Bennett G.S., Toyama Y., Kleinbart F., Holtzer H. (1981). Intermediate filament proteins in the developing chick spinal cord. Dev. Biol..

[59] Dent J.A., Polson A.G., Klymkowsky M.W. (1989). A whole-mount immunocytochemical analysis of the expression of the intermediate filament protein vimentin in *Xenopus*. Development.

[60] O’Hara C.M., Egar M.W., Chernoff E.A. (1992). Reorganization of the ependyma during axolotl spinal cord regeneration: Changes in intermediate filament and fibronectin expression. Dev. Dyn..

[61] Zamora A.J., Mutin M. (1988). Vimentin and glial fibrillary acidic protein filaments in radial glia of the adult urodele spinal cord. Neuroscience.

[62] Lazzari M., Franceschini V. (2001). Glial fibrillary acidic protein and vimentin immunoreactivity of astroglial cells in the central nervous system of adult *Podarcis sicula* (Squamata, Lacertidae). J. Anat..

[63] Morris R.J., Potten C.S. (1999). Highly persistent label-retaining cells in the hair follicles of mice and their fate following induction of anagen. J. Invest. Dermatol..

[64] Maeshima A., Yamashita S., Nojima Y. (2003). Identification of renal progenitor-like tubular cells that participate in the regeneration processes of the kidney. J. Am. Soc. Nephrol..

[65] Tumbar T., Guasch G., Greco V., Blanpain C., Lowry W.E., Rendl M., Fuchs E. (2004). Defining the epithelial stem cell niche in skin. Science.

[66] Kuwahara R., Kofman A.V., Landis C.S., Swenson E.S., Barendswaard E., Theise N.D. (2008). The hepatic stem cell niche: Identification by label-retaining cell assay. Hepatology.

[67] Morshead C.M., Reynolds B.A., Craig C.G., McBurney M.W., Staines W.A., Morassutti D., Weiss S., van der Kooy D. (1994). Neural stem cells in the adult mammalian forebrain: A relatively quiescent subpopulation of subependymal cells. Neuron.

[68] Codega P., Silva-Vargas V., Paul A., Maldonado-Soto A.R., DeLeo A.M., Pastrana E., Doetsch F. (2014). Prospective identification and purification of quiescent adult neural stem cells from their in vivo niche. Neuron.

[69] März M., Chapouton P., Diotel N., Vaillant C., Hesl B., Takamiya M., Lam C.S., Kah O., Bally-Cuif L., Strähle U. (2010). Heterogeneity in progenitor cell subtypes in the ventricular zone of the zebrafish adult telencephalon. Glia.

[70] Hendzel M.J., Wei Y., Mancini M.A., Van Hooser A., Ranalli T., Brinkley B.R., Bazett-Jones D.P., Allis C.D. (1997). Mitosis-specific phosphorylation of histone H3 initiates primarily within pericentromeric heterochromatin during G2 and spreads in an ordered fashion coincident with mitotic chromosome condensation. Chromosoma.

[71] Taupin P. (2007). BrdU immunohistochemistry for studying adult neurogenesis: Paradigms, pitfalls, limitations, and validation. Brain Res. Rev..

[72] Zacchetti A., Van Garderen E., Teske E., Nederbragt H., Dierendonck J.H., Rutteman G.R. (2003). Validation of the use of proliferation markers in canine neoplastic and non-neoplastic tissues: Comparison of KI-67 and proliferating cell nuclear antigen (PCNA) expression versus in vivo bromodeoxyuridine labelling by immunohistochemistry. APMIS.

[73] Cooper-Kuhn C.M., Kuhn H.G. (2002). Is it all DNA repair?: Methodological considerations for detecting neurogenesis in the adult brain. Dev. Brain Res..

[74] Bauer S., Patterson P.H. (2005). The cell cycle–apoptosis connection revisited in the adult brain. J. Cell Biol..

[75] Allen A.R., Smith G.T. (2012). Spinal transection induces widespread proliferation of cells along the length of the spinal cord in a weakly electric fish. Brain Behav. Evol..

[76] Blasko J., Martoncikova M., Lievajova K., Saganova K., Korimova A., Racekova E. (2012). Regional differences of proliferation activity in the spinal cord ependyma of adult rats. Cent. Eur. J. Biol..

[77] Lacroix S., Hamilton L.K., Vaugeois A., Beaudoin S., Breault-Dugas C., Pineau I., Lévesque S.A., Grégoire C.-A., Fernandes K.J.L. (2014). Central canal ependymal cells proliferate extensively in response to traumatic spinal cord injury but not demyelinating lesions. PLoS ONE.

[78] Johnson K., Bateman J., DiTommaso T., Wong A.Y., Whited J.L. (2018). Systemic cell cycle activation is induced following complex tissue injury in axolotl. Dev. Biol..

[79] Orts-Del’Immagine A., Wyart C. (2017). Cerebrospinal-fluid-contacting neurons. Curr. Biol..

[80] Zamora A.J. (1978). The ependymal and glial configuration in the spinal cord of urodeles. Anat. Embryol..

[81] Kútna V., Ševc J., Gombalová Z., Matiašová A., Daxnerová Z. (2014). Enigmatic cerebrospinal fluid-contacting neurons arise even after the termination of neurogenesis in the rat spinal cord during embryonic development and retain their immature-like characteristics until adulthood. Acta Histochem..

[82] Jalalvand E., Robertson B., Wallén P., Grillner S. (2016). Ciliated neurons lining the central canal sense both fluid movement and pH through ASIC3. Nat. Commun..

[83] Alibardi L. (1990). Electron microscopic observations on the myelination of the long-term regenerated caudal spinal cord in lizards and *Sphenodon*. Biol. Struct. Morphol..

[84] Simpson S.B., Duffy M. (1994). The lizard spinal cord: A model system for the study of spinal cord injury and repair. Prog. Brain Res..

[85] Alibardi L., Wibel R., Simpson S.B. (1993). Scanning electron microscopic observations on the central canal of the regenerating tail spinal cord in lizards. Boll. Zool..

[86] Alibardi L., Gibbons J., Simpson S.B. (1993). 3H-GABA administration during tail regeneration of lizards and autoradiographycal localization. J. Hirnforsch..

[87] Alibardi L. (2019). Cerebrospinal fluid-contacting neurons in the regenerating spinal cord of lizards and amphibians are likely mechanoreceptors. J. Morphol..

