# Supplementary material for: Radial Glia and Neuronal-like Ependymal Cells Are Present within the Spinal Cord of the Trunk (Body) in the Leopard Gecko (Eublepharis macularius)
Source: J Dev Biol. 2022 Jun 1;10(2):21. doi: 10.3390/jdb10020021 (PMC9224675; doi:10.3390/jdb10020021)
Supplement: Supplementary file 1 [file jdb-10-00021-s001.zip › jdb-1679181-supplementary.pdf]

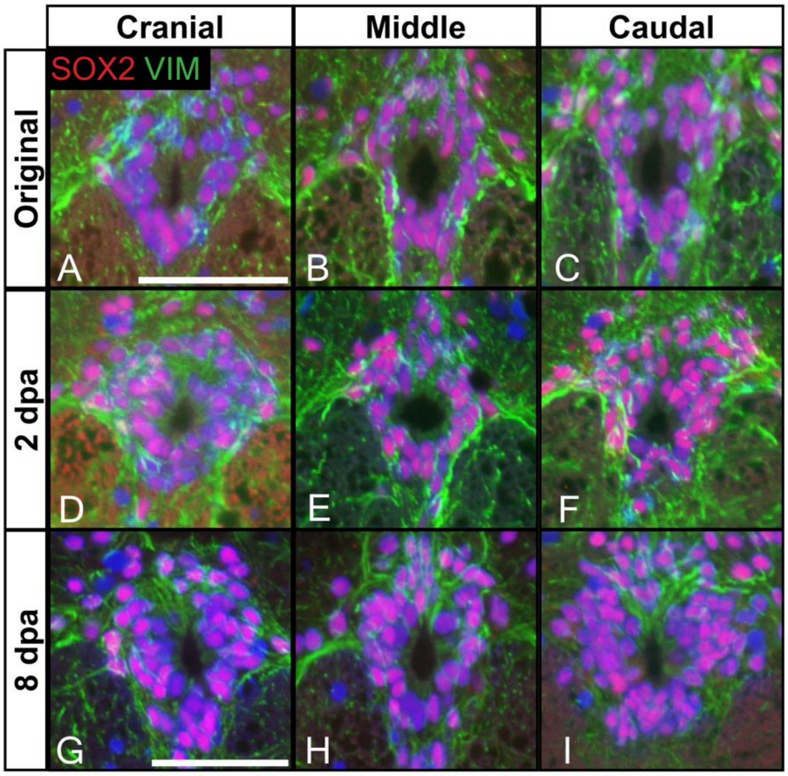

Figure S1. SOX2/Vimentin is expressed by the ependymal layer of the trunk spinal cord, and is unchanged after tail loss. (**A-C**) SOX2 and Vimentin are expressed in the ependymal layer in each segment of trunk spinal cord prior to tail loss. (**D-I**) Two days (**D-F**) and 8 days (**G-I**) after tail loss, ependymal cells continue to express SOX2 and Vimentin along the length of the body spinal cord. Note that expression remains relatively consistent across all three segments and time points. dpa=days post-autotomy. Scale bars: A-F, G-I=10μm.

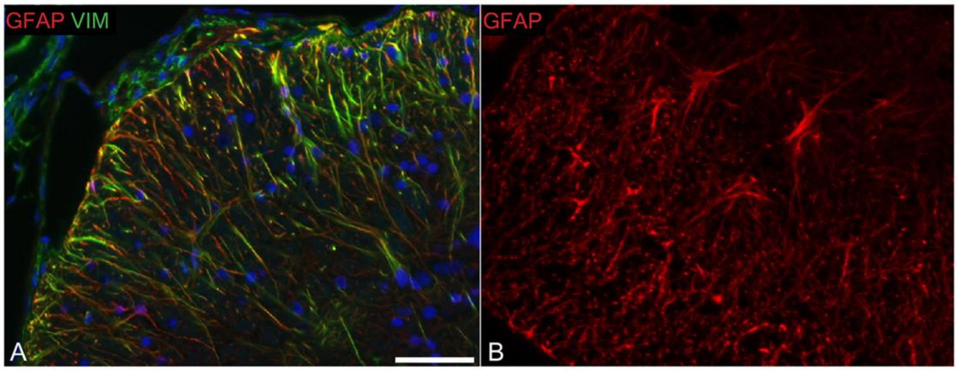

Figure S2. Radially-oriented GFAP+ processes and astrocytes are present within the white matter of the trunk spinal cord. (**A**) GFAP+ and Vimentin+ processes seen projecting radially toward the pial surface of the spinal cord in the dorsolateral white matter. While some processes are GFAP+/Vimentin+, others are GFAP+/Vimentin–. (**B**) GFAP+ astrocytes at the border of the grey and white matter in the ventral horns of the trunk spinal cord. Scale bar=10μm.

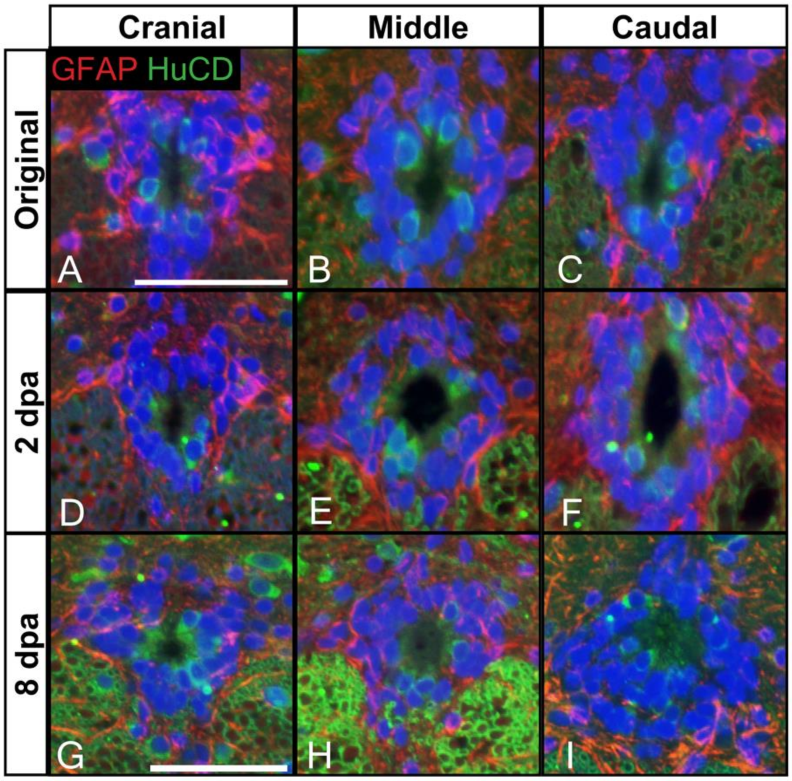

Figure S3. GFAP/HuCD is expressed by the ependymal layer of the trunk spinal cord, and is unchanged after tail loss. (**A-C**) GFAP and HuCD are expressed in the ependymal layer in each segment of trunk spinal cord prior to tail loss. (**D-I**) Two days (**D-F**) and 8 days (**G-I**) after tail loss, ependymal cells continue to express GFAP and HuCD along the length of the trunk spinal cord. Note that expression remains relatively consistent across all three segments and time points. dpa=days post-autotomy. Scale bars: A-F, G-I=10μm.
